# Classification and Analysis of Multiple Cattle Unitary Behaviors and Movements Based on Machine Learning Methods

**DOI:** 10.3390/ani12091060

**Published:** 2022-04-20

**Authors:** Yongfeng Li, Hang Shu, Jérôme Bindelle, Beibei Xu, Wenju Zhang, Zhongming Jin, Leifeng Guo, Wensheng Wang

**Affiliations:** 1 Agricultural Information Institute, Chinese Academy of Agriculture Sciences, Beijing 100086, China; yongfeng.li@student.uliege.be (Y.L.); shuhang@caas.cn (H.S.); xuxiaobei224@163.com (B.X.); zhangwenju@caas.cn (W.Z.); jinzhongming@caas.cn (Z.J.); 2 AgroBioChem/TERRA, Precision Livestock and Nutrition Unit, Gembloux Agro-Bio Tech, University of Liège, 5030 Gembloux, Belgium; jerome.bindelle@uliege.be

**Keywords:** behavior classification, inertial measurement units, machine learning, precision livestock farming

## Abstract

**Simple Summary:**

Traditionally, farmers are unable to pay enough attention to individual livestock. An increasing number of sensors are being used to monitor animal behavior, early disease detection, and evaluation of animal welfare. In this study, we used machine learning algorithms to identify multiple unitary behaviors and movements of dairy cattle recorded by motion sensors. We also investigated the effect of time window on the performance of unitary behaviors classification and discussed the necessity of movement analysis. This study shows a feasible way to explore more detailed movements based on the result of unitary behaviors classification. Low-cost sensors provide remote monitoring of animal behaviors to help producers comprehensively and accurately identify the health status of individual livestock in real-time.

**Abstract:**

The behavior of livestock on farms is the primary representation of animal welfare, health conditions, and social interactions to determine whether they are healthy or not. The objective of this study was to propose a framework based on inertial measurement unit (IMU) data from 10 dairy cows to classify unitary behaviors such as feeding, standing, lying, ruminating-standing, ruminating-lying, and walking, and identify movements during unitary behaviors. Classification performance was investigated for three machine learning algorithms (K-nearest neighbors (KNN), random forest (RF), and extreme boosting algorithm (XGBoost)) in four time windows (5, 10, 30, and 60 s). Furthermore, feed tossing, rolling biting, and chewing in the correctly classified feeding segments were analyzed by the magnitude of the acceleration. The results revealed that the XGBoost had the highest performance in the 60 s time window with an average F1 score of 94% for the six unitary behavior classes. The F1 score of movements is 78% (feed tossing), 87% (rolling biting), and 87% (chewing). This framework offers a possibility to explore more detailed movements based on the unitary behavior classification.

## 1. Introduction

Monitoring cattle behavior provides options to improve animal welfare, production, genetic breeding, and farm management. Observing individual animals based on farmers’ subjective experiences is challenging in large farming operations. Behaviors to monitor can be classified into two broad categories: unitary behaviors and movements [1]. Unitary behaviors can be defined as those activities that the animals perform only one at a time, not being able to perform two unitary behaviors simultaneously. These include grazing, ruminating, resting, etc. Usually, they hold a significant share of the time budget, although some behaviors, for example, drinking, can be short in duration. Each of these unitary behaviors can be composed of a repetition of several constituent movements such as chewing and swallowing while grazing. Additional non-constituent movements can be performed during unitary behaviors while not being essential to this behavior. One example of such non-constituent movement is fly-swatting, which can be performed during any unitary behavior. Automatic sensor monitoring systems have been used to objectively monitor the health status of individual animals, among which wearable sensors are most commonly developed and welcomed due to their real-time monitoring of cattle behaviors [2]. For example, sound sensors or noseband pressure sensors fixed on the head were used to recognize ruminating and feeding behaviors [3,4]. Three-axis accelerometers or inertial measurement units (IMU), including a gyroscope placed on the ear or the neck of cows, have been shown to allow the detection of unitary behaviors such as feeding, ruminating, standing, lying, and walking [5,6].

Accelerometers or IMU sensors can measure changes in force during the postural transition and local movement to classify unitary behaviors, and the duration and intensity of cattle unitary behaviors and movements can further predict significant events, e.g., calving, estrus, lameness, and disease. Benaissa et al. [7] developed a logistic regression model to predict both calving and estrus based on variables (e.g., lying time, number of steps, ruminating time, traveled distance) that were extracted from accelerometers (neck- and leg-mounted) and indoor localization sensors. Lameness was detected by monitoring the duration of lying, standing, and walking using leg-mounted accelerometers [8]. Gusterer et al. [9] identified the early disordered cows based on activity level and rumination recorded by a three-axis accelerometer.

To monitor the condition of cattle more accurately, a large number of studies have explored the factors that affect the classification of cattle behavior. Firstly, the worn position of the sensor is closely related to the behavior that can be monitored. Sensors fixed on cows’ leg was best for monitoring walking, lying, and standing behaviors [10]. The back was an effective position to monitor the lying (on the right or the left sides) and standing position and the transition between those two [11]. Necks and ears were found useful not only in the monitoring of overall posture but also for detecting head movements performed during feeding and ruminating [12,13]. Accelerometers attached to the tail allowed the prediction of calving time using the frequency and intensity of the tail raising [14]. Secondly, the sampling frequency of the sensor, the data segmentation method, and the algorithm selection also impact the classification performances [15,16].

Most research has focused on unitary behaviors and their constituent movements. However, some non-constituent movements are equally important from an animal welfare perspective. Stereotyped behaviors, defined as highly repetitive, invariant, functionless behaviors, may help reflect the welfare level of the individual and the herd [17]. Feed tossing, as a stereotyped behavior, can be classified as a non-constituent movement during feeding and was witnessed in approximately 10% of cows fed in elevated troughs, resulting in up to 5% feed wastage every week [18]. The medium-tie-stall system showed a higher percentage of feed tossing than the short-tie-stall system, but the reasons for feed tossing have never been clearly explained [19]. Non-constituent movements do not occur in every individual cow and always appear intermittently during unitary behaviors. It is worth noting that identifying more movements can help to gain a better understanding of the relationship between animals and their environment so as to improve animal welfare and production level. When cows are fed in the stall, two constituent movements are performed alternately, namely, lowering heads to roll feed into the mouth with the tongue and raising the head to chew. Compared with cows with heads held horizontally, cows eating with their heads down produce 17% more saliva, which affects feeding efficiency and rumen function [18]. Unlike the biting during grazing, in which cattle need to tear the forage forcefully, cattle in the barn only need to use their tongues to roll the feed into their mouths [20,21]. In addition, the recognition of detailed movements during feeding is conducive to more accurate and direct monitoring of feed intake. Microphones and noseband pressure sensors have been used to monitor jaw movement and classify biting and chewing [22,23]. The duration of these two movements is closely related to feed intake [24].

In this paper, a new cattle behavior classification framework was proposed to explore more movements based on the classification of unitary behaviors. To this end, cows’ motion data was recorded via the IMU sensors (three-axis accelerometer and three-axis gyroscope) fixed on the neck. Six unitary behaviors were labeled with synchronized recorded videos, including feeding, standing, lying, ruminating-standing, ruminating-lying, and walking. A total of 13 features were extracted over four time windows with 50% overlap, and three machine learning algorithms were developed to classify unitary behaviors. Furthermore, the correctly predicted feeding segments were analyzed to recognize the movement of feed tossing, rolling biting, and chewing during feeding using the thresholds of acceleration.

## 2. Materials and Methods

### 2.1. Animals and Experiment Arena

The study was carried out on a commercial dairy farm from 24 July to 1 August 2021 in Cao County, Heze City, Shandong Province, China. The research methods and use of animals in the experiments were approved by the Chinese Academy of Agricultural Sciences for experimental animal welfare and ethics (IAS2021-220). Fifty Holstein cows were housed in a free-stall barn with a length of 90 m and a width of 15 m. A total of 10 healthy lactating Holstein-Friesian cows were selected to attach in-house-designed IMU-based collars, and each cow was continuously monitored for at least seven hours during the experiments. The cows were milked at 8:30 am, 4:30 pm, and 12:00 pm every day. Seven cameras (Hikvison DS-2CE16G0T-IT3, Hikvison, Hangzhou, China) covered 90% of the barn area to record cattle behaviors, and video recording time and sensor time were synchronized. For the convenience of the observation, only day’s (08:30 a.m.–4:30 p.m.) data and videos were used for analysis in this paper.

### 2.2. Sensor System

**Figure 1:**
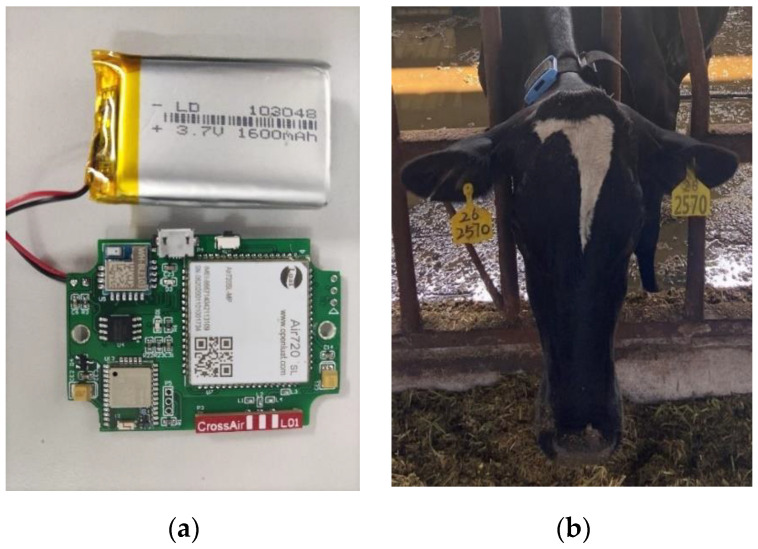
Sensor internal structure and battery (**a**), The position of the sensor on the cow’s neck (**b**).

The sensor device consists of a microprocessor, a Micro-Electro-Mechanical System (MEMS) IMU (MPU-6050, InvenSense, San Jose, CA, USA), a 4G transmission module, a GPS, and a wrapped battery (3.7 v, 1600 mAh) by a waterproof plastic box (Figure 1a). MPU6050 was chosen as the IMU in this work, which was composed of a 3-axis accelerometer (±4 g) and a 3-axis gyroscope (±2000 °/s). This low-cost IMU was widely used in wearable devices due to its low power consumption and high performance. The sampling frequency was set to 32 Hz (32 rows per second). To prevent the collision with the headlock, the sensor was fixed on the neck right behind the right ear (Figure 1b), and the weight below the neck was used to keep the sensor in place. The collected sensor data, sample time, device id, battery voltage, latitude, and longitude were encoded into hexadecimal and transmitted to the server using a 4G network every 40 s.

### 2.3. Data Collection

The experiment was conducted in an actual production environment, and the behavioral data that were obscured by other cows or not observed and were difficult to define were all eliminated in the study. Six unitary behaviors were defined by referring to the previous research [6], including feeding, standing, lying, ruminating-standing, ruminating-lying, and walking. One trained observer labeled the behavior in the videos based on the definition in Table 1. The dataset included 400 individual behavior segments (each segment more than 30 s), with a total of approximately 78.6 h. The dataset was unbalanced, with only 2% of the data labeled as “walking” behavior (Table 2). In addition to the unitary behaviors, Table 1 also presents the descriptions of movements of feed tossing, rolling biting, and chewing during feeding.

### 2.4. Unitary Behavior Classification and Analysis

**Figure 2:**
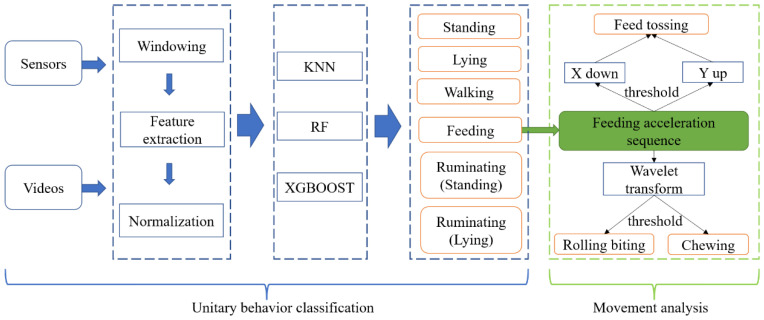
Process of unitary behavior classification and movement analysis. KNN: K-nearest neighbors; RF: Random forest; XGBoost: Extreme boosting algorithm.

The overall processing flow for behaviors classification and analysis is provided in Figure 2. The framework can be divided into two stages: (1) long segments that were continuous and undivided during unitary behaviors and (2) the movements of the feeding segments correctly classified in the first step. Feature extraction, model construction, and analysis were all implemented using python 3.7.

#### 2.4.1. Data Preprocessing

**Figure 3:**
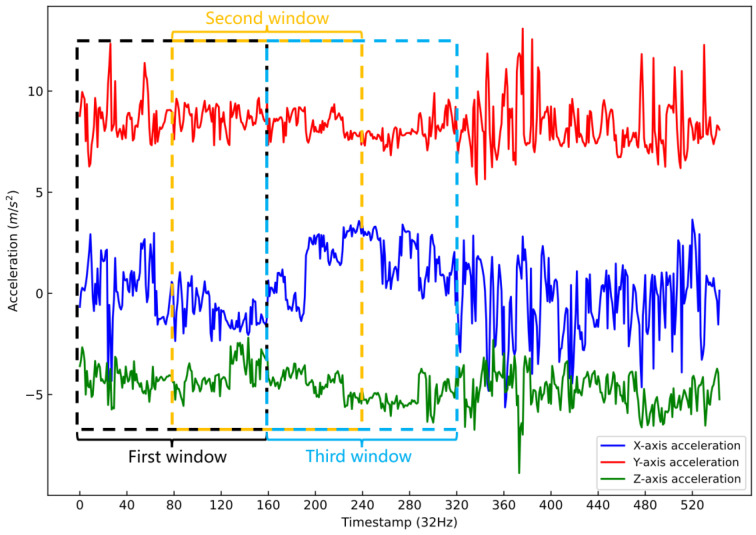
Example of a sliding 5 s time window with a 50% overlap.

Six time series data columns and a behavior label column were acquired from motion sensors and videos, respectively. Figure 3 shows the sliding time window (Δt=5 s) applied to the time series data with a 50% overlap. A single behavior label was contained in each time window. Four window sizes and the total sample size are shown in Table 3. Both time domain and frequency domain were considered for calculating features. The time-domain features included maximum, minimum, mean, variance, standard deviation, peak-to-peak distance, and mode in a certain time window. The frequency-domain features were calculated after the Fast Fourier Transform for the time series, including direct current, power spectral density, spectral entropy, and spectral energy. In addition, overall dynamic body acceleration (ODBA) and movement variation (MV) were also calculated based on previous research [25,26]. Finally, the values of all the thirteen features were normalized to the 0–1 range.

#### 2.4.2. Machine Learning Algorithms

According to the stratified sampling method, 70% of each type of behavior was selected as the training set and the remaining 30% as the testing set. A 10-fold cross-validation method was adopted to obtain the parameters of each model. Three machine learning algorithms were used to classify cattle unitary behavior. The same dataset was applied to the three algorithms to fairly compare the classification performance.

1. K-nearest neighbors (KNN)

The rationale of the KNN algorithm is that each sample can be represented by the K nearest neighbors [27]. The classification for samples can be calculated by the distance between the testing sample and the training sample. This distance generally uses Euclidean distance or Manhattan distance. According to the order of distance, the testing sample is classified as the closest class to it. In order to measure the sample distance on the same scale, all features are normalized, and then the selection of K value is obtained through cross-validation.

2. Random forest (RF)

The RF is a bagging ensemble algorithm [28]. RF trains a base classifier (cart decision tree) by randomly selecting some samples (with replacement) and extracting several features each time. The Gini coefficient is used to evaluate the effect of cart splitting, and all base learners vote to produce the final prediction. The weight of the base learner is the same, and thus the expectation of the model is approximately equal to that of a single base learner, and the variance is gradually reduced [29].

3. Extreme boosting algorithm (XGBoost)

The XGBoost is a boosting ensemble algorithm that efficiently implements the Gradient boosting decision tree algorithm [30]. The base learner is one kind of simple decision tree. The initial model is trained to fit the residual between the predicted value and the actual value of the model from the previous round. The penalty term is added to the loss function to prevent the model from overfitting.

#### 2.4.3. Movement Analysis

**Figure 4:**
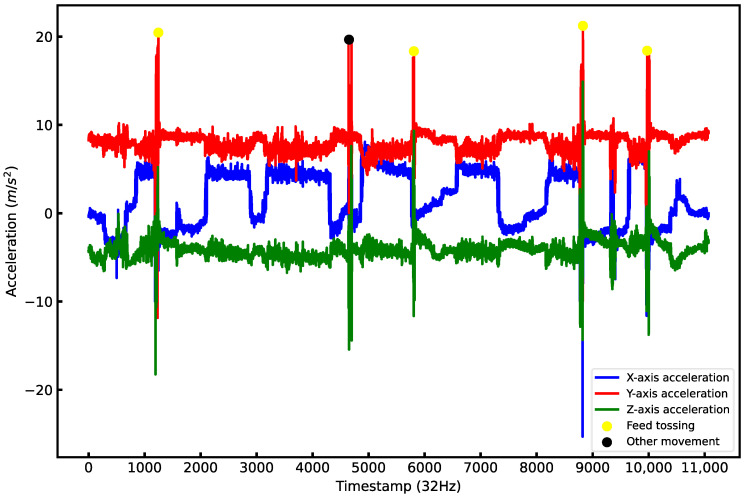
Alternate occurrence of rolling biting and chewing on the feeding interval and feed tossing after rolling biting.

When cows are fed in the barn, they usually perform rolling biting and chewing alternately. Specifically, cows lowered their heads to the bottom of the feed and rolled the feed into their mouths, relying on their tongues, and thereafter cows raised their heads up to chew for a while. The motion sensors fixed along the cows’ necks were parallel to the ground when they stood or lay down in a normal resting position. When cows lowered their heads to roll the feed into their mouths, their necks were at an acute angle to the ground. Because the motion sensor was affected by the gravity component, the X-axis value rose rapidly and remained steady at around 6 $m/s^{2}$; When cows chewed with their heads rising off the ground, their necks returned parallel to the ground, and the X-axis value dropped rapidly to near 0 $m/s^{2}$ (Figure 4). In addition, some cows suddenly raised their heads from one side at the end of the rolling biting and threw the feed into the air even on their backs, in which both X-axis and Y-axis increased drastically and then decreased rapidly after reaching the peak. The yellow dots in Figure 4 show the time point of feed tossing, which generally happened right after the rolling biting. The black dot represents the increase in accelerations caused by other strenuous head movements (such as repeated rapid head shaking) during chewing.

The results of the machine learning model were used as the input for detailed movement analysis (Figure 2). In the movement analysis, feeding segments were successfully predicted by the model and merged according to timestamps. Each segment interval lasted between 10 and 50 min. Since feed tossing did not occur for each cow in all feeding segments, which were randomly selected for movement analysis, two thresholds were used to identify feed tossing: threshold A of the X-axis acceleration, below which the X-axis acceleration suddenly dropped, and threshold B of the Y-axis acceleration, above which the Y-axis acceleration became a local maximum at the same time. The feed tossing could be recognized when both thresholds were reached at the same time (Figure 4). Threshold A and threshold B were 1 $m/s^{2}$ and 12.5 $m/s^{2}$, respectively. In addition, to identify rolling biting and chewing intervals, wavelet transform filtering was used to remove the details (high-frequency information) and only retain the approximation of the X-axis acceleration (low-frequency information). Threshold C of the X-axis acceleration and threshold D of the length of time interval was used to distinguish rolling biting and chewing. The X-axis acceleration curve after filtering was denoted as h(t), the intersection of h(t) and y=threshold C was denoted as $P\;\left\{p_{t_{1}},p_{t_{2}}\cdots p_{t_{i}}\right\}$, and the time interval between $p_{t_{i-1}}$ and $p_{t_{i}}$ was denoted as d. Therefore, if $h\left(\frac{t_{i}+t_{i-1}}{2}\right)>threshold\;C$ and d>threshold D, d belonged to rolling biting; if $h\left(\frac{t_{i}+t_{i-1}}{2}\right)<threshold\;C$ and d>threshold D, d belonged to chewing. Threshold C was the mid-range of X-axis acceleration, which was the arithmetic mean of the maximum and minimum. Threshold D was 1 s, and times of rolling biting and chewing less than 1 s were removed.

### 2.5. Evaluation of the Prediction

Each machine learning model on the same testing set was considered to evaluate the classification performance of the model. Precision (Pr), sensitivity (Se), and F1 score were calculated by the following formulae:

(1) $$\mathrm{Pr}=\frac{\mathrm{TP}}{\left(\mathrm{TP}+\mathrm{FP}\right)}$$

(2) $$\mathrm{Se}=\frac{\mathrm{TP}}{\left(\mathrm{TP}+\mathrm{FN}\right)}$$

(3) $$F1\;\mathrm{score}=2\times \frac{\mathrm{Pr}\times \mathrm{Se}}{\mathrm{Pr}+\mathrm{Se}}$$

where true positive (TP) is the number of samples for which the behavior was predicted by the model and observed in the video; false negative (FN) is the number of samples for which the behavior was observed in the video but not correctly predicted; false positive (FP) is the number of samples for which the behavior was predicted by the model but not observed in the video.

## 3. Results

### 3.1. Unitary Behavior Classification

The classification performance shown in Table 4 indicated that the precision and sensitivity of all three algorithms in all behaviors exceeded 81% when the time window was greater than 30 s. With a window of 60 s, the XGBoost algorithm had the highest precision and sensitivity for feeding (96%, 96%) and ruminating-lying (94%, 91%). In addition, the sensitivity of KNN (94%) in classifying walking was higher than that of RF (83%) and XGBoost (89%). Finally, the highest precision (88%) for standing was yielded using XGBoost with a window size of 30 s.

**Figure 5:**
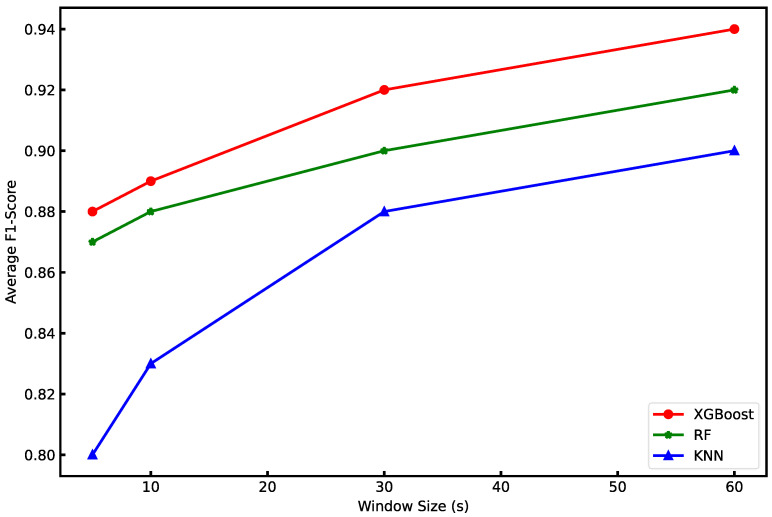
Average F1 scores of the three algorithms across four time windows.

The comparison of the average F1 score of the three algorithms across four time windows is shown in Figure 5. The best average F1 score (94%) was obtained using the XGBoost algorithm in the 60 s time window. In every time window, the average F1 score for the XGBoost algorithm is higher than the RF and KNN. The average F1 score increased monotonically with window size. Compared to the 5s time window, the average F1 score of KNN, RF, and XGBoost in the 60 s time window improved by 10%, 5%, and 6%, respectively.

### 3.2. Detailed Movement Analysis

Detailed movement analysis used randomly selected consecutive feeding segments that were correctly classified in Section 3.1. The timestamp of the feed tossing movement was detected in seconds. The start time and end time of alternate rolling biting and chewing were recorded, respectively. The number of movements recognized by the model was greater than the actual observed by the video. The precision of identifying feed tossing movement was the lowest (69%), and the sensitivity was the highest (89%) (Table 5). The F1 score for rolling biting and chewing shared the value of 87% (Table 5).

## 4. Discussion

A variety of methods have been proposed to improve cattle behavior classification, including feature extraction methods, combinations of different types of sensors, selection of sensor locations, and optimization of sampling frequencies [31]. Pressure sensors and sound sensors may be able to obtain more details of jaw movements, but it is difficult to obtain overall body movement information (e.g., lying, standing, walking). To reduce the burden on cattle caused by wearing too many sensors, only one IMU neck-motion sensor was used to monitor cattle behavior. The aim of this paper was not only to recognize unitary behaviors by using the neck-mounted IMU sensor and machine learning algorithms but also to further investigate the movements and patterns in detailed feeding movements.

In previous studies, the acceleration amplitude of the classification behavior fixed on the neck was usually set to ±2 g [32,33]. This amplitude is sufficient for unitary behaviors (feeding, ruminating, resting, etc.). However, some large movements would exceed this range, such as feed tossing, head bumping, and so on [34]. In this study, the scale ranges of the accelerometer and gyroscope were configured as ±4 g and ±2000 °/s, respectively. This was done to ensure that all behaviors and movements were completely recorded.

The size of the time window is known to have an impact on the performance of cattle behavior classification and computational load on an embedded system [35]. The greater the length of the time window, the fewer samples are used for model training, which may result in a negative impact on model performance. However, the features calculated in a larger time window are more representative of fixed behaviors. The results in the paper show that the increase in the time window size improves the classification performance, which was consistent with the study of Daniel Smith [12]. A large number of behavior transitions are included in the process of data segmentation, resulting in a fixed time window containing two different behavioral data [36]. The overlap of the time window is good for the prediction of transitory behavior transitions [16]. Therefore, it is beneficial to long-duration unitary behavior classification by appropriately increasing the moving time window size. In addition, the dynamic time window may be suitable for some shorter behaviors (drinking, walking, etc.) in time [35,37]. The fixed time window has no effect on short-duration sharp (feed tossing) or frequent changes (rolling biting and chewing) movements.

The performance of the classification is also affected by the movement intensity and movement part of behavior. Feeding, ruminating, and lying is classified based on activity level and variations [32,38]. Generally, the activity level for each behavior is sorted as follows: feeding and walking > rumination (standing and lying) > standing and lying. Due to similar activity levels, standing and walking are sometimes misclassified as lying and feeding, respectively. There is also a risk of misclassification for movements happening in the same part during these unitary behaviors; for example, ruminating while standing and lying are often misclassified because both movements of the jaw are similar. In addition, due to strong breathing, the standing may be misclassified as rumination in cattle under hot weather.

Recognizing more behaviors can help increase the overall classification accuracy and, in turn, support a more comprehensive evaluation of animal welfare [39]. However, most relevant works are still limited to the recognition of unitary behaviors. Stereotyped behaviors are often performed when cattle are fed at housing farms but are never observed at open pastures. The occurrence of these behaviors may be caused by the boredom of the environment, which indicates that the environment should be changed [40]. Feed tossing, a stereotyped behavior, frequently occurred in housing situations, directly leading to feed waste. To the best of our knowledge, this is the first study to recognize feed tossing by IMU sensor, providing an effective tool for monitoring feed wasting and further evaluating the relationship between animals and the environment. It should be noted that feed tossing may not occur in dairy barns with properly designed feeding lanes, but that the technique used to identify feed tossing may be useful for the detection and prediction of other short-term behaviors and movements.

Moving time window is difficult to use for detecting movements that change suddenly and frequently. The duration of feed tossing is very short, and therefore, it is difficult to detect over a time window lasting several seconds. In the case of rolling biting and chewing movements, a fixed time window can hardly contain one single movement since they appear in time alternately. Given that all the above-mentioned movements occur during feeding, the correctly identified feeding segments were used for further analysis, with the sudden changes in the acceleration along the X-axis and the Y-axis used to identify feed tossing, and the X-axis after the wavelet transform used to identify rolling biting and chewing.

The identification process of feed tossing is different from the detection of unitary behaviors. Unitary behaviors distinguish the differences between each behavior through features, while feed tossing is to find out the timestamp of abnormal data from feeding time segments. Threshold A was used to determine that the feed tossing appeared at the end of rolling biting with the head down. Threshold B was defined as the critical point of feed tossing. If the threshold B was too high, some feed tossing would not be detected. If it was too small, other behaviors would be mistaken for feed tossing. Irrelevant movements interfered with the recognition of feed tossing. As shown in Table 5, the number of feed tossing predicted by the model was higher than what was observed in the video, which was due to the misclassification of some similar movements, such as rapid head shaking and head bumping with other cows during feeding [18]. Some feed tossing events were not recognized because the amplitude of the movement was too small.

Feed intake can be estimated directly based on understanding the characteristic constituent movements during feeding. There is a difference in eating behavior between cattle raised in houses and those raised on pastures. When cattle eat in the stall, the duration of rolling biting with the head down is related to the amount of feed. If the amount of feed is low, less feed is rolled into the mouth by the tongue each time, resulting in more rolling biting bouts of longer duration. The data analysis in the paper indicates that the movements of cows while feeding in the barn can be recognized by the amplitude of acceleration based on head movements. Threshold C was used to divide the feeding segments into rolling biting and chewing. The acceleration value was only considered in relation to the ups and downs of the head, but the acceleration frequency was not considered. Therefore, threshold D was used to exclude some short-term head shaking and feed searching, which would lead to misclassification. In the future, unsupervised classification algorithms can be used to improve the accuracy of discriminating rolling biting from chewing.

The paper only analyzed the detailed movements in the feeding behavior, and other unitary behaviors also include a variety of constituent movements and non-constituent movements. For example, tongue playing during feeding or lying, lying posture transition, and heavy breathing during standing and lying, all of which are mixed into long-term unitary behaviors but are vital to improving the breeding environment and animal welfare. Our research suggested that it is a feasible way to classify the unitary behaviors first and then find the detailed movements from the focused behavior segments. In the future, sensors can be used to monitor more behaviors and movements, and independent samples collected continuously from other farms should be validated to improve the robustness and scalability of the model.

## 5. Conclusions

In this paper, a new framework was proposed for cattle behaviors classification via neck-worn IMU sensors in two steps: (1) unitary behaviors classification and (2) detailed movement analysis. This study showed that IMU sensors were not only able to distinguish unitary behaviors but also effectively identify non-constituent (feed tossing) and constituent movement (rolling biting and chewing) during unitary behaviors. All unitary behaviors were better recognized on the three algorithms (KNN, RF, and XGBoost) as the time window increased. The XGBoost algorithm had the best unitary behaviors classification performance under the 60 s time window with an average F1 score of 94%. The F1 score of feed tossing, rolling biting, and chewing were 78%, 87%, and 87%, respectively. It provided a new potential framework to monitor animal behavior and movement more comprehensively and improve farm management and animal welfare. Future research could be considered to demonstrate that more behaviors and detailed movements (e.g., tongue playing, excessive rub) could be recognized with this framework. Combining IMU time series and GPS location data will also provide insight into animal behavior in open pastures.

## Figures and Tables

**Table 1:** Description of unitary behaviors and detailed movements during feeding.

	Class	Description
Unitary behaviors	Feeding	The animal puts its head into the stall and eats in the feeding rack
	Standing	The animal stands without head movement and rumination
	Lying	The main body touches the cubicle floor without head movement and rumination
	Ruminating-standing	The animal regurgitates food bolus from the rumen, chews and swallows it while standing
	Ruminating- Lying	The animal regurgitates food bolus from the rumen, chews, and swallows it while lying
	Walking	The animal moves in one direction for at least 30 s
Movements during feeding	Feed tossing	The animal takes a mouthful of feed then throws the feed into the air or even over its back by twisting the neck
	Rolling biting	The animal lowers its head and uses its tongue to roll feed into the mouth during feeding
	Chewing	The animal chews feed with its head up during feeding

**Table 2:** The number of segments, proportions, and total time for each behavior.

Behavior	Number of Segments	%	Duration (HH:MM:SS)
Feeding	76	27	21:19:15
Standing	87	11	08:26:28
Lying	90	19	15:06:13
Ruminating-standing	58	18	14:03:16
Ruminating-lying	54	23	17:57:04
Walking	35	2	01:44:22
Total	400	100	78:36:38

**Table 3:** Sample size of unitary behavior when each time window (5 s, 10 s, 30 s, and 60 s) overlaps by 50%.

Window Size	Sample
5 s	116,960
10 s	58,474
30 s	19,485
60 s	9739

**Table 4:** Precision (Pr) and sensitivity (Se) {%} for each unitary behavior (F: feeding; S: standing; L: lying; RS: ruminating-standing; RL: ruminating-lying; W: walking) and machine learning algorithms across four time windows. KNN: K-nearest neighbors; RF: Random forest; XGBoost: Extreme boosting algorithm.

Machine Learning Algorithm		Time Window							
	Unitary Behavior	5 s		10 s		30 s		60 s	
		Pr	Se	Pr	Se	Pr	Se	Pr	Se
KNN	F	85	80	89	84	92	92	94	96
	S	77	80	77	80	84	83	85	86
	L	85	84	88	83	89	87	91	84
	RS	70	73	74	78	81	84	87	86
	RL	80	81	83	86	90	90	87	92
	W	82	83	82	85	89	88	100	94
RF	F	86	88	89	89	92	92	95	96
	S	83	84	81	86	86	89	83	91
	L	94	90	93	90	91	92	95	90
	RS	82	81	84	82	86	83	92	86
	RL	89	91	90	93	92	93	91	95
	W	92	81	92	82	98	82	100	83
XGBoost	F	88	89	91	91	94	94	96	96
	S	84	85	82	86	88	91	85	93
	L	93	91	94	91	93	94	96	91
	RS	84	81	86	84	90	87	94	91
	RL	90	92	92	95	94	96	93	96
	W	91	83	91	87	97	88	100	89

**Table 5:** The number of in the actual observed and model predicted for three movements. Precision (Pr), sensitivity (Se), and F1 score (F1) (%) for three movements (FT: feed tossing; RB: rolling biting; C: chewing) were analyzed.

Movement	Pr	Se	F1	Actual Observed	Model Predicted	True Positive
FT	69	89	78	127	184	114
RB	86	88	87	446	518	392
C	87	89	87	460	529	409

## Data Availability

The data presented in this study are available on request from the corresponding author.
